# Exploring the Influence of Family Attitudes and Individual Psychological Factors on Antibiotic Utilization: A Pilot Study

**DOI:** 10.3390/healthcare12121213

**Published:** 2024-06-18

**Authors:** Paola Castellano, Paolo Maria Russo, Michela Mazzetti

**Affiliations:** Department of Medical and Surgical Sciences, Alma Mater Studiorum University of Bologna, 40138 Bologna, Italy; p.russo@unibo.it (P.M.R.); michela.mazzetti@unibo.it (M.M.)

**Keywords:** antibiotic abuse, antibiotic adherence, individual factors, family model, psychological well-being

## Abstract

The inappropriate use of antibiotics gives rise to detrimental consequences, both physical and emotional, with a decreased quality of life and higher levels of anxiety and depression. The current observational study aimed to investigate the association between awareness, beliefs, and behaviors toward antibiotics, highlighting the modulating role of individual and psychological factors in response to illness and medication. Through an online questionnaire, several psychological indexes, as well as knowledge of, attitude toward, and experiences with antibiotics, both individual and family-related, were assessed in a sample of 100 responders (74 females, mean age 33.37 ± 11.36). A positive association between intake behavior, awareness, and individual attitude emerged. Familial approach to antibiotics appears as a predictor of individual attitude and behavior toward these drugs, and awareness about antibiotic risks mediate the relationship between the tendency to be more compliant with prescriptions (R^2^ = 0.300; MSE = 1.541; F(2, 98) = 20.737; *p* < 0.0001). Moreover, individuals with a personality characterized by higher conscientiousness are more aware of antibiotic risks (*p* < 0.01), whereas individuals with a lower awareness are those with higher indexes of psychophysical discomfort (i.e., anxiety, perceived stress, somatization) and levels of emotional rebound (*p* < 0.05). Anxiety (F(3, 96) = 3.874; *p* = 0.012; R^2^ = 0.108) and somatization (F(2, 97) = 3.114; *p* = 0.030; R^2^ = 0.089) also significatively moderated the intake behavior, despite the family approach. Overall, the current study provides preliminary findings regarding the way in which family experiences and individual psychological aspects may be influencing factors in the behavior and attitude towards antibiotics and can be used to plan patient-centered therapeutic communication and education.

## 1. Introduction

Therapeutic adherence, referring to the extent to which individuals follow medical prescriptions [1], is a crucial factor for resolving infectious diseases treated with antibiotics and preventing serious consequences for both individuals and the environment [2,3]. The lack of adherence, characterized by excessive consumption, improper timing, and a tendency towards self-medication, contributes to antibiotic resistance—bacteria’s ability to resist antibiotic action [4]. Medication use is part of “illness behavior”, a coordinated set of adaptive behavioral changes in response to illness [5]. This behavior is influenced by psychological and social aspects, as demonstrated by studies emphasizing individual factors and the family’s role in healthcare practices. Addressing the first type of effects, several studies indicate that personality and emotions influence adherence to medical prescriptions, both for chronic conditions [6,7,8] and short-term antibiotic treatments [9]. Referring to specific personality effects, individuals exhibiting greater cooperation and conscious behavior are generally more compliant, whereas those experiencing higher levels of anxiety and worry tend to show lower compliance [10].

Additionally, as COVID-19 studies have recently reported, fear of illness and health anxiety can lead to incorrect medication behaviors, including self-medication and antibiotics overuse [11,12], probably due to an overestimation of an efficacy in unnecessary conditions [13]. Indeed, widespread lack of knowledge concerning antibiotics and their associated risks contributes to incorrect antibiotic intake [14], aligning with the low knowledge and awareness reported in the World Health Organization’s 2015 survey across 12 countries [15]. On the other hands, anxiety and fear about one’s health can induce the opposite results, leading individuals to avoid medications due to perceived unnatural composition of the drugs and potential adverse effects [16].

Beyond and in interaction with individual factors, the family environment also plays a crucial role in shaping individuals’ health behaviors [17,18], acting as a setting for health promotion or inhibition [19]. It influences health outcomes [20] and engagement in unhealthy practices like, for example, smoking [21] and drug consumption [22].

To the best of our knowledge, there is limited data available regarding the connection between the perceived family model and individual attitudes towards antibiotics. The majority of studies have concentrated on patients’ emotional responses [23] and the communication styles of physicians [24], with no investigation delving into the modulating role of personality and anxiety.

In order to understand the complexity of the factors involved and effectively guide physician–patient communication and awareness campaigns aimed at the proper use of antibiotics, a multifactorial approach is necessary. This approach should be able to unveil the interactive effects of individual/emotional factors, family models, and attitudes on illness behaviors, in accordance with the theory of planned behavior [25,26]. This theoretical model posits that human behavior is guided by three kinds of considerations, i.e., behavioral belief (about the likely consequences and experiences associated with the behavior), normative beliefs (about the normative expectations of significant others), and control beliefs (about the presence of factors facilitating or obstructing the performance of the behavior).

The family model can be regarded as a source of behavioral and normative beliefs, while personality traits and anxiety may be associated with the control attitudes.

Given these overarching assumptions, the present study is aimed to examine whether exposure to a family model characterized by an appropriate, rejecting, or improper attitude towards antibiotics influences the conscious use, rejection, or overuse of this medication, considering in addition the modulating role of personality and of emotional health, namely anxiety, perceived stress, and somatization.

For this purpose, we conducted a pilot study, administering a series of questionnaires to a sample of the general population and investigating whether the relationship between antibiotic intake and family model is indeed modulated by personality factors and emotional well-being. Subsequently, we performed a series of moderation and mediation analyses (see Section 2.4), with the family model as the independent variable and actual antibiotic intake behaviors as the dependent variables.

In particular, we expected the family model, as an example of normative belief, to influence awareness regarding antibiotics and actual illness-related behavior. Additionally, we expected that family influence could be modulated by individual factors such as conscientiousness and openness, which should promote awareness and correct behavior, and by psychophysical discomfort (i.e., a high level of anxiety, somatization tendency, and perceived stress), which might encourage antibiotic overuse or refusal, even if necessary.

## 2. Materials and Methods

### 2.1. Participants

Participants were recruited online through a link shared on major social networks and by word of mouth. Consistent with this type of data collection, the link was active for a limited period of three months, and the sample size was not determined in advance. Individuals were invited to take part in a study aimed at analyzing the use of medicines in the general population (inclusion criteria: 18 to 50 years of age, with the objective of not increasing the within-group variability of the data; native Italian speakers). Participants were informed that they should complete a questionnaire comprising psychological and health-related questions. Data collection took place from July 2022 to September 2022.

### 2.2. Procedure

Upon clicking on the provided link, participants read and signed the informed consent. They were then directed to complete a series of questions, including assessments of sociodemographic information (e.g., gender, age, qualification, occupation, marital status), validated questionnaires for personality traits, and evaluations of symptoms related to anxiety, perceived stress, and somatization.

Additionally, participants responded to a set of ad hoc questions exploring the approach of their family of origin toward antibiotics. The questionnaire also included a revised version of the “Antibiotic Resistance: Multi-Country Public Awareness Survey”, created by the World Health Organization [15].

### 2.3. Questionnaires

#### 2.3.1. Assessment of Individual Traits

A battery of validated questionnaires was employed to assess specific individual traits, including personality traits (Big Five Questionnaire; BFQ-R) [27]; the anxiety trait, intended as a stable and long-standing condition of worry (Trait Scale of the State-Trait Anxiety Index; STAI-Y2) [28]; perceived stress, intended as a measure of the degree to which life situations are appraised as stressful (Perceived Stress Scale; PSS) [29]; somatization, intended as the interaction between somatic and psychological problems (Psychosomatic Problem Scale; PSP) [30]. The clear explanation of each questionnaire is provided in Section S1 of the Supplementary Materials.

#### 2.3.2. Assessment of Practice, Knowledge, and Awareness about Antibiotics and Antibiotic Resistance

An adapted form of the “Antibiotic Resistance: Multi-Country Public Awareness Survey”, created by the World Health Organization [15], was utilized. The revised survey comprised a total of 26 items.

The first part included two questions regarding the amount of antibiotics taken recently (number of antibiotic cycles taken in the last year/last month) and four questions about good practices regarding antibiotic intake (individual score: sum of correct answers).

The second part comprised nine statements designed to assess participants’ knowledge of antibiotic resistance, with responses recorded categorically (True/False). Individual scores were determined by summing the correct answers. “Knowledge” refers to levels of understanding regarding the issue of antibiotic resistance. One example item was: “Antibiotic resistance occurs when your body becomes resistant to antibiotics and they no longer work as well” (False).

The final section included ten statements concerning beliefs and attitudes regarding antibiotic use and resistance to assess participants’ overall (antibiotic) awareness. Responses were collected using a 5-point Likert scale (1 = strongly disagree, to 5 = strongly agree), and the total scores obtained by the sum were used as individual scores. “Awareness” refers to levels of (self)awareness and understanding regarding ways to address the problem of antibiotic resistance. One example item was: “People should use antibiotics only when they are prescribed by a doctor or nurse”.

Finally, an additional ad hoc question was included to investigate participants’ past compliance with antibiotic prescriptions. The item was, “In the past, I have always taken antibiotics following the prescriptions”, and responses were provided on a numerical rating scale ranging, ranging from 0 = totally false for me, to 10 = totally true for me.

#### 2.3.3. Assessment of Individual and Family Approach to Antibiotics

Eight ad hoc questions were introduced to explore participants’ families of origin and their approach to antibiotics. This distinguished between a family model that used antibiotics appropriately (i.e., avoiding auto-prescription and medication and respecting dosage regimen), one that rejected antibiotics, and another that used antibiotics improperly. Examples of items include: “If I had to take antibiotics, my family ensured I followed the doctor’s orders” (appropriate, two questions), “My family of origin was against the use of antibiotics” (rejection, three questions), and “In my house, it was easy to find antibiotics left over from previous treatments” (improper, three questions). Responses were given on a numerical rating scale ranging from 0 (totally false for me) to 10 (totally true for me). The total scores within each family model were used as an individual indicator.

### 2.4. Data Analysis

Preliminary assessments were conducted to ensure there were no violations of the assumptions of normality and homogeneity of variance.

Descriptive analyses and ANOVAs were employed to characterize the sample and assess potential covariates. Correlation analyses were performed to examine relationships between variables of interest.

To further explore the relationship between the family model (independent variable) and the antibiotics taken (dependent variables), considering the influence of psychological indexes as moderator variables, a series of multiple moderation analyses, with 10,000 accelerated bootstrap samples and bias-corrected confidence intervals, were conducted.

Additionally, to better understand the role of awareness about antibiotics (mediator variable) in the relationship between the family model (independent variable) and previous compliance to prescriptions (dependent variables), a mediation analysis, with 10,000 accelerated bootstrap samples and bias-corrected confidence intervals, was performed.

All statistical analyses were conducted using IBM SPSS Statistics 28.

## 3. Results

A total of 100 participants (74 females and 26 males, aged between 18 and 50, with a mean age of 33.37 ± 11.36 years) participated in the study. The sociodemographic and individual characteristics of the sample are presented in Table 1.

Preliminary analyses found no significant differences for antibiotics consumption in the last year and past compliance with prescriptions for gender and age.

### 3.1. Correlational Analyses: Family Examples, Individual Characteristics, and Illness Behavior

Correlation analyses showed that having had a family of origin which rejected antibiotics was negatively correlated to the quantity of antibiotics taken in the last year (*p* < 0.05) (see Table S1 in Supplementary Materials). On the contrary, having had a family of origin which used antibiotics improperly was positively correlated to the amount of antibiotics taken (*p* < 0.05), and negatively correlated both to the knowledge of good practices of antibiotics intake (*p* < 0.01) and to the awareness about antibiotics. The latter was positively correlated to a family of origin with an appropriate approach to and behavior with antibiotics, as well for a previous good compliance (*p* < 0.01) (see Table S1 in Supplementary Materials). 

Also, significant correlations emerged between psychological facets and antibiotic behavior. As expected, the personality traits of conscientiousness, agreeableness, and openness were positively correlated with antibiotics awareness (*p* < 0.01) (see Table S2 in Supplementary Materials). The agreeableness trait was also in positive correlation with past compliance to prescriptions (*p* < 0.05) (see Table S2 in Supplementary Materials). Regarding indexes of psychological well-being, anxiety, perceived stress, and somatization were negatively correlated to awareness (*p* < 0.05) (see Table S2 in Supplementary Materials).

### 3.2. Moderation Analyses: The Moderating Role of Emotional Well-Being

The moderating roles of anxiety, somatization, and perceived stress were examined, using the family model as the independent variable and behavior towards antibiotics as the dependent variable.

The level of anxiety was found to significantly moderate the rejection of antibiotics observed within one’s own family, as indicated by the model (F(3, 96) = 3.874; *p* = 0.012; R^2^ = 0.108) and the interaction of the (demonizing) family model with anxiety (b = 0.003; t(96) = 2.581; *p* = 0.011).

**Figure 1:**
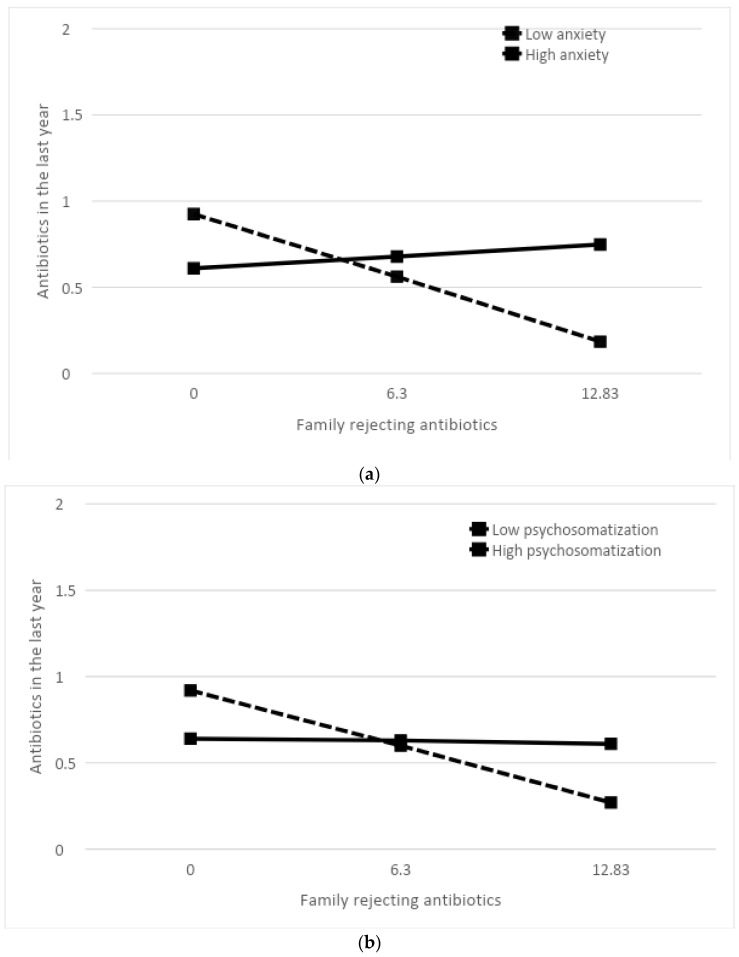

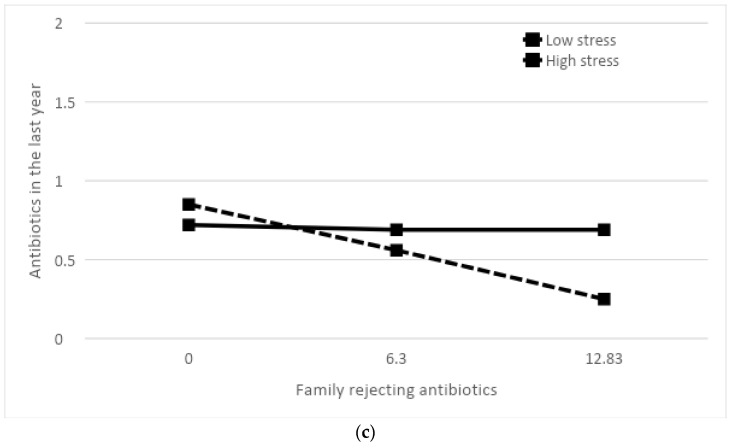
Moderation models between family approach, antibiotic intake, and anxiety (**a**), somatization (**b**), and stress (**c**). Graphs show slopes of (**a**) anxiety, (**b**) somatization, and (**c**) stress predicting antibiotics intake at low, mean, and high levels of a family of origin negative toward antibiotics. When plotting the graphs, data automatically generated by the PROCESS analysis algorithm are used to classify low, mean, and high levels of continuous variables.

As shown in Figure 1a, among individuals with a high level of anxiety, the family’s demonization did not impact the amount of antibiotics taken in the last year. Conversely, for individuals with a low level of anxiety, antibiotic consumption was higher in low demonizing compared to high demonizing families (b = −0.058, t(96) = −3.380, *p* = 0.001).

In addition, the moderating role of somatization was also found to be significant (see Figure 1b), as evidenced by the model (F(2, 97) = 3.114; *p* = 0.030; R^2^ = 0.089) and the interaction with the family model (b = 0.004; t(96) = 2.119; *p* = 0.037). Similar to the results for anxiety, somatization moderated antibiotic intake only within low demonizing families: individuals with a high level of somatization exhibited drug consumption independent of family (whether high or low) demonization. In contrast, among individuals with low anxiety, antibiotic intake was higher in low demonizing compared to high demonizing families (b = −0.050, t(96) = −3.043, *p* = 0.003).

Finally, the same analysis conducted with perceived stress as the moderation variable (see Figure 1c) did not yield a significant model (F(2, 97) = 2.520; *p* = 0.063; R^2^ = 0.073).

### 3.3. Mediation Analysis: The Mediating Role of Awareness

**Figure 2:**
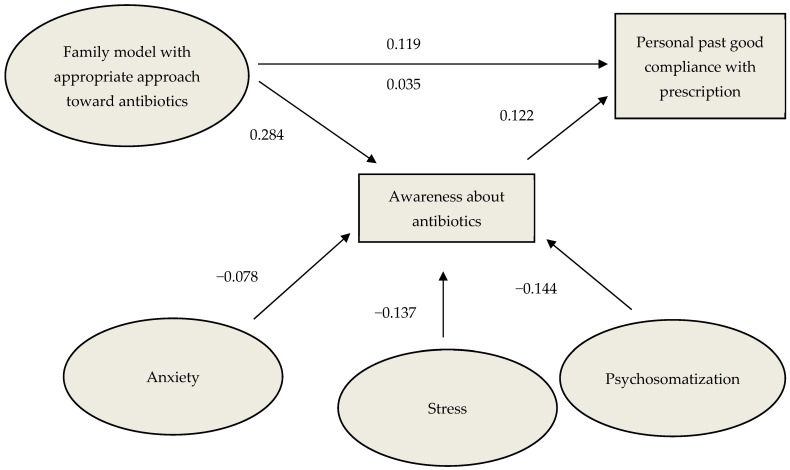
Mediation model between antibiotic awareness, family approach, compliance, and psychological indexes. Having had a family with an appropriate approach to antibiotics is associated with a better past compliance with antibiotic prescription, and this relationship is in part mediated by an individual’s awareness about antibiotics. Standardized coefficients, with associated 95% confidence intervals, are provided for direct effects (above arrow line), as well as for indirect effects (below arrow line). Higher levels of anxiety, perceived stress, and somatization are associated with a reduced antibiotics awareness (i.e., the potentiality to use antibiotics in a less-conscious way).

Then, to further investigate the relationship between an individual’s past compliance with antibiotic prescription (dependent variable) and an appropriate family model toward antibiotics (independent variables), under the influence of awareness about antibiotics (mediator variable), a mediation analysis was performed, yielding significant results (R^2^ = 0.300; MSE = 1.541; F(2, 98) = 20.737; *p* < 0.0001). That is, the more appropriate the approach of the family of origins towards antibiotics, the higher the respect for a doctor’s prescription. A strong positive relationship between an individual’s awareness about antibiotics and past compliance was also evidenced, as well as, through a series of regression analysis, the predictive role of anxiety, stress, and somatization toward awareness about antibiotics (see Figure 2 for standardized coefficients of direct, total, and indirect effects, together with associated 95% confidence intervals).

## 4. Discussion

The present study examined the role of the family of origin and dispositional psychological aspects in antibiotic intake behavior. To our knowledge, this is the first study which combines family-related experiences with antibiotics and individual personality traits, as well as psychological well-being (i.e., levels of anxiety, perceived stress, and somatization), with the aim of identifying how they affect individuals’ approaches to antibiotics.

Consistent with our predictions, the attitude of the family of origin was associated with the quantity of antibiotics taken during the past year. Specifically, individuals whose families have demonized antibiotics tend to consume fewer antibiotics, but also to be less compliant with medical prescriptions when there is an actual need, whereas individuals from families that have improperly used antibiotics tend to consume more, with a lower level of awareness and reduced knowledge about proper usage. As expected, individuals from families that have used antibiotics correctly demonstrate greater compliance with prescriptions and a higher awareness of antibiotic usage and associated risks. Our data align with the well-established Biobehavioral Family Model (BBFM) [31], a multilevel systemic biopsychosocial model that posits that family relationships modulate an individuals’ psychophysiological development and health behaviors [31,32,33].

Indeed, attitudes acquired during childhood seem to be reiterated, and this aspect is noteworthy. Attitude plays a fundamental role in the action process, as postulated by the theoretical framework of the Theory of Planned Behavior (TPB) by Ajzen [25,26], which has been applied in several studies to predict correct health behaviors [34,35], including medication adherence (e.g., melanoma [36]; diabetes [37]; asthma [38]). Our data correspond with the same trend, demonstrating how the family example and the normative beliefs stemming from it are indeed capable of shaping the illness-related behaviors exhibited in adulthood. However, this does not appear to be the sole force at play in understanding (and, for professionals, contributing to modifying) the attitudes and behaviors of the population. The current research also investigated the role of dispositional personality traits and indices of psychological well-being in regards to antibiotic behavior. As expected, and in line with a body of literature [7,10,39,40], individuals with a personality characterized by higher levels of conscientiousness, openness to experience, and agreeableness tend to be more aware of antibiotic functioning and risks. However, the results were intuitive, in contrast to what might be expected concerning psychological health. Indeed, there are two controversial positions concerning the role of health-related fear and anxiety. One position is more connected to hypochondria and reflects individuals’ fears of illness [41], leading patients to engage in incorrect health behaviors as coping strategies, including excessive healthcare utilization [42] and misuse of medications such as antibiotics [13,43]. On the other hand, emotions can deter individuals from taking medicines, even when prescribed correctly, often due to the fear of potential side effects [16]. Furthermore, health anxiety, as well as health-related beliefs and behaviors, may be influenced by familial influences and passed down from parents to children [44,45].

In this study, psychophysical discomfort, characterized by increased levels of anxiety, stress, and a tendency to somatization, leads to reduced awareness of antibiotic risks and acts as a mediator in the relationship between family history of correct antibiotic usage and individual compliance with prescriptions. This finding about psychological discomfort suggests that the less conscious use of antibiotics may serve as a coping strategy to combat the fear of illness. Moreover, anxiety alone appears to be a risk factor for antibiotic consumption, as highlighted by the moderation model that includes a family of origin that has rejected and demonized antibiotics and recent antibiotic intake. If being exposed to a family model that opposes the use of antibiotics leads to reduced antibiotic consumption, this is not the case when high levels of anxiety are present, as the quantity of antibiotics taken does not vary significantly based on the family’s negative approach. The same pattern holds true for somatization, suggesting that the tendency to experience physical symptoms of illness influences antibiotic intake more than does the family’s influence.

## 5. Limitations

The present study is not exempt from limitations that warrant caution in interpreting the results. Firstly, the achieved sample size is limited and needs to be increased to reach a size that allows for age-stratified analysis, enabling verification of whether and how the balance between family influence, individual traits, and perceived distress has changed over time and across generations.

Secondly, this investigation was conducted on the general population through retrospective data collection and utilized self-report indices. The obtained data will need to be compared with the directly observed behavior of patient groups experiencing conditions where antibiotic use is necessary or contraindicated. This evaluation will assess the actual therapeutic adherence and its covariation with family background, personality, and psychophysical well-being/discomfort.

Finally, it should be considered that some of the tools utilized were specifically constructed for the present research, albeit partially based on the WHO survey [15].

## 6. Implications

With these caveats in mind, however, we find the potential practical implications of these data to be relevant. This pertains both to the management of the direct doctor-patient relationship and the implementation of campaigns for antibiotic use awareness and the prevention of antimicrobial resistance.

Indeed, in the medical patient practice, it appears absolutely necessary to consider individual psychological characteristics and the degree of psychological well-being or distress, in addition to the family example and the level of apparent awareness: anxiety can nullify the effect of the family example, in both directions. Awareness is therefore a key vector, which in turn is influenced by psychological well-being, the tendency to somatize, and the anxiety one may experience in the face of symptoms already present or feared to appear in the prognosis or as side effects of pharmacological treatment.

Similarly, in awareness/education campaigns, it is necessary to provide information that is not only easily understandable and memorable but also capable of providing reassurance (and reducing anxiety): increasing control/knowledge in turn increases the likelihood of reacting to stress (illness) with active and adaptive coping strategies, in line with Ajzen’s model [25,26], which posits that the more positive the attitude and subjective norm, and the greater the perceived control, and the stronger the individual’s intention to engage in the behavior.

## 7. Conclusions

With the caution warranted by the sample size of what should be considered a pilot study, the reported results shed light on a central aspect of this study: what individuals learn as children plays a role in illness behavior. The familiar pattern is re-proposed in individual choices: individuals whose families have demonized antibiotics tend to use them less, whereas those with families of origin that have used antibiotics in an improper manner tend to consume them more; moreover, individuals whose families have used antibiotics correctly tend to be more compliant with doctors’ prescriptions. But importantly, the example acquired as children is modulated by the level of psychophysical distress and anxiety, in particular. Therefore, the ultimate goal is to provide prescribers with an awareness of the need to address the negative emotions of patients, considering the affective, cognitive, and emotional regulation components of their illness behavior. Certainly, the small sample size and the use of (partially) non-validated tools to investigate individual and family approaches to antibiotics constitute limitations in this research. Nevertheless, future studies should continue to search for suitable tools to support doctors in patient care, focusing on long-term and holistic health, encompassing drugs, therapy, beliefs, and psychological well-being. In summary, just as families can play a role in education and prevention by teaching the correct use and the necessity of adhering to prescriptions, so too must doctors and informational campaigns act on two distinct levels, cognitive (awareness), and, especially in the dyadic relationship, emotional, reassuring the patients and addressing their anxiety associated with therapeutic choices.

## Figures and Tables

**Table 1:** Participants’ baseline characteristics for all the investigated variables (sociodemographic, psychological, antibiotic-related).

Variable	N; M ± SD
Gender	74 F, 26 M
Age (M ± SD)	33.37 ± 11.36
Marital status	54 Unmarried, 30 Married, 11 Cohabitating, 3 Divorced, 2 Widowed
Qualification	6 Middle School, 37 High School, 46 Degree, 11 PhD/Postgraduate Specialization
Occupation	31 Student, 31 Employee, 6 Freelance, 5 Retired, 4 Unemployed, 23 Other
BFQ-Openness (M ± SD)	3.53 ± 0.64
BFQ-Conscientiousness (M ± SD)	3.58 ± 0.63
BFQ-Extraversion (M ± SD)	3.15 ± 0.54
BFQ-Agreeableness (M ± SD)	3.53 ± 0.52
BFQ-Neuroticism (M ± SD)	3.19 ± 0.76
STAI-Y2 (M ± SD)	46.52 ± 12.61
PSS (M ± SD)	19.27 ± 7.93
PSP (M ± SD)	20.20 ± 6.23
Family Antibiotic Rejection	6.30 ± 6.53
Family Antibiotic Appropriate Use	17.27 ± 3.95
Family Antibiotic Improper Use	14.86 ± 7.39
Antibiotics taken in the last month *	92 Never, 5 Once, 2 Twice
Antibiotics taken in the last year *	52 Never, 32 Once, 16 Twice
Knowledge of good intake practices	4.91 ± 1.15
Knowledge of antibiotic resistance	13.64 ± 1.35
Awareness about antibiotics	40.40 ± 4.45
Past compliance	9.08 ± 1.46

Notes: * cycle of therapy. M = Males; F = Females; BFQ = Big Five Questionnaire; STAI-Y2 = State-Trait Anxiety Index—trait measure; PSS = Perceived Stress Scale; PSP = Psychosomatic Problem Scale.

## Data Availability

Data are contained within the article and Supplementary Materials.

## Supplementary Materials

The following supporting information can be downloaded at: https://www.mdpi.com/article/10.3390/healthcare12121213/s1, Table S1: Correlations between family models and antibiotic practice, knowledge, and awareness; Table S2: Correlations between psychological indexes (i.e., personality traits, anxiety, perceived stress, and somatization), and antibiotic practice, knowledge, and awareness.
