# Supplementary material for: Exploring the Influence of Family Attitudes and Individual Psychological Factors on Antibiotic Utilization: A Pilot Study
Source: Healthcare (Basel). 2024 Jun 18;12(12):1213. doi: 10.3390/healthcare12121213 (PMC11202973; doi:10.3390/healthcare12121213)
Supplement: Supplementary file 1 [file healthcare-12-01213-s001.zip › healthcare-2887581-supplementary.pdf]

SUPPLEMENTARY MATERIALS

Table S1. Correlations between family models and antibiotic practice, knowledge and awareness.

	Antibiotics/ last year	Antibiotics/ last month	Antibiotics Intake Knowledge	AMR knowledge	Antibiotics awareness	Past compliance
Family Antibiotic Rejection	-0.206*	0.146	-0.127	-0.142	-0.014	-0.095
Family Antibiotic Appropriate Use	-0.080	0.019	0.160	0.152	0.252*	0.414**
Family Antibiotic Improper Use	0.223*	0.180	-0.352**	-0.126	-0.313**	-0.131

Notes: *p < 0.05; ** p < 0.01. AMR = Antimicrobial resistance.

Table S2. Correlations between psychological indexes (i.e., personality traits, anxiety, perceived stress and somatization) and antibiotic practice, knowledge and awareness.

	Antibiotics/las year	Antibiotics/ last month	Antibiotics Intake Knowledge	AMR knowledge	Antibiotics awareness	Past compliance
BFQ-N	0.016	-0.076	-0.018	-0.147	-0.081	-0.095
BFQ-E	0.248*	0.085	0.090	-0.015	0.169	-0.022
BFQ-C	0.134	-0.096	-0.039	-0.042	0.282**	0.189
BFQ-A	0.089	0.004	0.188	0.244*	0.323**	0.253*
BFQ-O	0.042	-0.043	0.178	0.201*	0.218*	0.039
STAI-Y2	0.043	-0.009	-0.165	-0.051	-0.221*	-0.130
PSS	0.052	0.022	-0.165	-0.079	-0.244*	-0.029
PSP	0.024	0.101	-0.080	-0.117	-0.202*	-0.112

Notes: *p < 0.05; ** p < 0.01. AMR = Antimicrobial resistance. BFQ = Big Five Questionnaire; N = Neuroticism; E = Extraversion; C = Conscientiousness; A = Agreeableness; O = Openness to Experience; STAI-Y2 = State-Trait Anxiety Index – trait measure; PSS = Perceived Stress Scale; PSP = Psychosomatic Problem Scale.

1. Psychological validated questionnaires

1.1 Big Five Questionnaire (BFQ-R; Caprara et al., 1993)

The Italian version of the Big Five Questionnaire (BFQ-R; Caprara et al., 1993) was used to assess personality traits of Openness, Conscientiousness, Extraversion, Agreeableness and Neuroticism. The scale includes 60 items, and the answers are provided on a 4-point Likert scale (from 1 = Absolutely false for me to 4 = Absolutely true for me). Example of items are: "I am always informed about what is happening in the world" (Openness), "I tend to be very thoughtful" (Conscientiousness), "I seem to be an active and vigorous person" (Extraversion), "If necessary, I don't shy away from giving help to strangers" (Agreeableness), "My mood is subject to frequent fluctuations" (Neuroticism). Internal consistency of the total scale in the present study was α = .87.

1.2 State-Trait Anxiety Index (STAI-Y2) (Spielberg et al., 1983)

The Italian version of the trait scale of the State-Trait Anxiety Index (STAI-Y2) (Spielberg et al., 1983) was administrated to evaluate the level of anxiety trait of participants. The scale includes 20 items, and the answers are provided on a 4-point Likert scale (from 1 = Almost never to 4 = Almost always). An example of item is: "I worry too much about things that don't really matter". Internal consistency in the present study was α = 0.92.

1.3 Perceived Stress Scale (PSS; Cohen et al., 1994)

The Italian version of the Perceived Stress Scale (PSS; Cohen et al., 1994) was used to assess the level of stress and discomfort perceived by participants during the last month. The scale includes 10 items, and the answer are provided on a 5-point Liker scale (from 0 = Never to 4 = Very often). An example of item is: "In the last month, how often have you felt like you were unable to control the important things in your life?". Internal consistency of the scale in the present study was α = 0.86.

1.4. Psychosomatic Problem Scale (PSP; Hagquist, 2009)

The Italian version of the Psychosomatic Problem Scale (PSP; Hagquist, 2009) was administered to evaluate the amount of physical symptoms experienced by participant during the last six months. The scale includes 8 items, and the answers are provided on a 5-point Likert scale (from 1 = Never to 5 = Always). An example of item is: "Did you feel dizzy?". Internal consistency of the scale in the present study was α = 0.76.
